# Adsorption Properties of β- and Hydroxypropyl-β-Cyclodextrins Cross-Linked with Epichlorohydrin in Aqueous Solution. A Sustainable Recycling Strategy in Textile Dyeing Process

**DOI:** 10.3390/polym11020252

**Published:** 2019-02-02

**Authors:** José A. Pellicer, María I. Rodríguez-López, María I. Fortea, Carmen Lucas-Abellán, María T. Mercader-Ros, Santiago López-Miranda, Vicente M. Gómez-López, Paola Semeraro, Pinalysa Cosma, Paola Fini, Esther Franco, Marcela Ferrándiz, Enrique Pérez, Miguel Ferrándiz, Estrella Núñez-Delicado, José A. Gabaldón

**Affiliations:** 1 Dpto. de Ciencias de la Salud., Universidad Católica San Antonio de Murcia (UCAM), Avenida de los Jerónimos s/n, 30107 Guadalupe, Murcia, Spain; japellicer@ucam.edu (J.A.P.); mirodriguez@ucam.edu (M.I.R.-L.); mifortea@ucam.edu (M.I.F.); clucas@ucam.edu (C.L.-A.); mtmercader@ucam.edu (M.T.M.-R.); slmiranda@ucam.edu (S.L.-M.); vmgomez@ucam.edu (V.M.G.-L.), enunez@ucam.edu (E.N.-D.); 2 Universita degli Studi “Aldo Moro” di Bari, Dip. Chimica, Via Orabona, 4, 70126 Bari, Italy; paola.semeraro@uniba.it (P.S.); pinalysa.cosma@uniba.it (P.C.); 3 Consiglio Nazionale delle Ricerche CNR-IPCF, UOS Bari, Via Orabona, 4, 70126 Bari, Italy; pfini@ba.ipcf.cnr.it; 4 Biotechnology Department, Textile Industry Research Association (AITEX), Plaza Emilio Sala, 1, 03801 Alcoy, Spain; efranco@aitex.es (E.F.); mferrandiz@aitex.es (M.F.); 5 Colorprint Fashion, SL, Avda. Fco. Vitoria Laporta 104, 03830 Muro de Alcoy, Alicante, Spain; eperez@colorprintfashion.com (E.P.); produccion@colorprintfashion.com (M.F.)

**Keywords:** adsorption, Direct Red, β-CDs, HP-β-CDs, kinetics, isotherm, pulsed light

## Abstract

β-cyclodextrin (β-CD) and hydroxypropyl-β-cyclodextrin (HP-β-CD) were used to prepare insoluble polymers using epichlorohydrin as a cross-linking agent and the azo dye Direct Red 83:1 was used as target adsorbate. The preliminary study related to adsorbent dosage, pH, agitation or dye concentration allowed us to select the best conditions to carry out the rest of experiments. The kinetics was evaluated by Elovich, pseudo first order, pseudo second order, and intra-particle diffusion models. The results indicated that the pseudo second order model presented the best fit to the experimental data, indicating that chemisorption is controlling the process. The results were also evaluated by Freundlich, Langmuir and Temkin isotherms. According to the determination coefficient (*R*^2^), Freunlich gave the best results, which indicates that the adsorption process is happening on heterogeneous surfaces. One interesting parameter obtained from Langmuir isotherm is *q_max_* (maximum adsorption capacity). This value was six times higher when a β-CDs-EPI polymer was employed. The cross-linked polymers were fully characterized by nuclear magnetic resonance (NMR), Fourier transform infrared spectroscopy (FTIR), thermal gravimetric analysis (TGA). Also, morphology and particle size distribution were both assessed. Under optimized conditions, the β-CDs-EPI polymer seems to be a useful device for removing Direct Red 83:1 (close 90%), from aqueous solutions and industrial effluents. Complementarily, non-adsorbed dye was photolyzed by a pulsed light driven advanced oxidation process. The proposed methodology is environmental and economically advantageous, considering the point of view of a sustainable recycling economy in the textile dyeing process.

## 1. Introduction

Nowadays, the textile dyeing industry is one of the most polluting all over the world [1]. It is estimated that the apparel industry uses over five trillion liters of water per year [2], being the effluents derived from textile treatment and dyeing, responsible of 20% of freshwater pollution [3]. The direct toxic impact of dyes and their metabolites on different living organisms, including humans have been evidenced [4,5]. Due to their xenobiotic effects and persistence, azo dyes like Direct Red 83:1 has lasting disturbing effects on ecosystems. Even though these ecosystems have their natural ways for potential remediation, the process not always yields non-toxic or less toxic degradation products [6]. In fact, some environmental processes promote the transformation of harmless dyes into toxic metabolites, such as aromatic amines, diazonium and nitrenium ions, and hydrolyzed products [7]. In addition, the microflora of the skin or the gut ecosystem of mammalians transforms certain non-toxic dyes into toxic carcinogenic products [8]. These end-products further impart toxicity at different levels of biological compartments via different pathways [9]. Furthermore, the recurring disposal of dyes in aquatic ecosystems has caused serious and specific pollutions characterized by coloration visually unpleasant [10] and high chemical oxygen demand (COD), which impairs photosynthesis [11]. As consequence, efforts are being made to develop methods to avoid that dyes and their metabolites enter ecosystems.

Although physico-chemical methods seem to be effective in azo dyes decolorization [12,13], nowadays, their efficacy to detoxify is still questionable, being a global priority the development of alternative eco-friendly and cost-effective methods. There are many kinds of treatments used to remove dyes from wastewater, above all membrane separation [14], adsorption [15], electrochemical technologies [16], advanced oxidation processes (AOP) [17], and biodegradation [9,18]. Currently, the use of adsorbents material has been considered one of the finest treatments, due to its good performances, such as high efficiency without secondary pollution, ease handle and low running costs [19,20], being commonly classified as chemical and physical sorption. The first one involves chemical associations between the dye and the adsorbent, which is generally a consequence of the exchange of electrons and is generally irreversible [21]. The later kind implies feeble interactions, such as van der Waals forces, hydrogen bonds, polarity and dipole-dipole interactions [22].

The most commonly used adsorbent to eliminate polluting agents from aqueous solutions is activated carbon, due to its porous structure, but is expensive and with poor removal of dyes [19]. In addition, its thermal regeneration requires high energy consumption and sorption capacity could not completely restore [23]. Consequently, their use for water treatment at industrial scale is restricted and the same limitation applies to other commercial adsorbents, such as activated alumina, synthetic polymer resins and zeolites [24,25,26]. Thus, the development of novel economic, efficient, eco-friendly, and widely available adsorbents is required [27]. From this stand point, the interest in carbon-based adsorbents, such as polyethylene glycol-modified graphene oxide [28], gluten flour cross-linked graphene oxide composites [29] and cyclodextrins (CDs), have achieve major attention in water treatment in the last years [30,31,32] by several relevant advantages. Firstly, the CD molecules are polymers formed by the action of an enzyme on starch and can be currently synthesized at low price from natural resources. Secondly, the presence of hydroxyl groups, as well as their well-defined structures bestows CDs high reactivity and outstanding selectivity towards different dyes. Thirdly, it can be regenerated and reused after saturation because of their chemical stability. Fourthly, CDs are biodegradable and renewable; hence they would not cause further pollution. CDs are water soluble materials; therefore, it must be insolubilized before use in water treatment on an insoluble matrix or cross-linked. Epichlorohydrin (chloromethyl oxirane C_3_H_5_ClO, abbreviated EPI) is the most frequently used cross-linker [32]. It contains a chloroalkyl moiety and a highly reactive epoxide group [33], which under alkaline conditions, can effectively bind with hydroxyl groups. Thus, EPI can react with CDs yielding cross-linked networks and/or react with itself, yielding polymerized chains. The procedure transforms CD from water-soluble monomer into hydrophilic, insoluble three-dimensional polymer while keeping intact the cavity structure of CDs, which allows the formation of inclusion complexes with many different molecules [34,35].

Dye adsorption by CDs is not 100% efficient; therefore, unadsorbed amounts of dyes can contaminate the environment when released to it. The treatment of remaining amounts of dyes mixing them with hydrogen peroxide and subjecting this mixture to pulsed light (PL) technology has been proved to be an efficient strategy for dye degradation [17], with the ultimate goal of reducing the pollutant charge of wastewaters. In this kind of AOP, hydroxyl radicals that can degrade the dye molecule are generated from the photolytic split of hydrogen peroxide, due to the UV light generated by the light pulse.

The overall aim of this work was to investigate the potential of EPI cross-linked β-cyclodextrin **(**β-CD) and hydroxypropyl-β-cyclodextrin (HP-β-CD) as adsorbents of Direct Red 83:1, for water treatment applications. Firstly, the effects of contact time, and initial pH and dye concentration, as well as the regeneration condition for the used adsorbents, and the possible adsorption mechanism was explored. Secondly, the experimental data obtained were fitted to various kinetic and isotherm models and compared with previous studies found in the literature. Thirdly, a thorough characterization of both polymers was carried out using the Fourier transform infrared spectroscopy (FTIR), nuclear magnetic resonance (NMR) and thermal gravimetric analysis (TGA). Also, the morphology of the samples was assessed by scanning electron microscopy. Finally, the efficiency of the PL technology on dye decolorization was followed by spectrophotometry.

## 2. Materials and Methods

### 2.1. Chemicals

The cyclodextrins used (β- and HP-β-CDs) were from AraChem (Tilburg, The Netherlands), sodium borohydride (98%), sodium hydroxide (98%), epichlorohydrin (99%), acetone (99.5%) and hydrogen peroxide (30%) were purchased from Sigma-Aldrich (Madrid, Spain). Direct Red 83:1 (95% purity) was from Colorprint (Alcoy, Spain).

### 2.2. Preparation of the Polymers and Dye Solutions

The CD-EPI polymers were synthesized following the method described by Pellicer et al. (2018) [36], which is a modification of the protocol of Renard et al. (1997) [37]. In brief, NaBH_4_ and each CD were mixed in water, and then, a NaOH solution and EPI were sequentially added and mixed until the polymer becomes formed. This was washed with acetone and dried overnight.

Solutions of the azo dye Direct Red (CAS 90880-77-6, C_33_H_20_N_6_Na_4_O_17_S_4_, molecular weight (MW): 992.77) were prepared in the range of 25–300 mg/L, in order to evaluate the adsorption capacity of the polymers [36].

### 2.3. Analyses and Data Evaluation

The concentration of dye was measured in the supernatant using a spectrophotometer (Shimadzu UV-1603), after the equilibrium was reached. The absorbance was measured before and after the treatment with the polymers at the peak absorbance of the dye (λ_max_ = 526 nm; ε_526_ = 1065 M^−1^cm^−1^) [36].

### 2.4. Adsorption Experiments

The adsorption experiments were measured at 25 °C, using solutions containing a known concentration of dye, ranging from 25 to 300 mg/L that were mixed with a known quantity of adsorbent polymer for a selected time. Then, the polymer was removed from the solution. In each experiment, 1 g of polymer was added to 50 mL of dye solution. The mixture was stirred at a fixed speed of 500 rpm. Every 10 min, it was measured the remaining concentration of dye in the solution, until the equilibrium was reached, and the maximum adsorption capacity was obtained. The samples were centrifuged at 4000 rpm for 5 min prior to measuring the concentration of dye in the solution. The experiments were carried out in triplicate.

### 2.5. Polymer Characterization

Fourier transform infrared spectroscopy (FTIR) spectra, ^1^H-NMR spectra, thermogravimetric analysis (TGA) and derivative thermogravimetric (DTG) were performed in order to characterize the polymer as reported before [36]. In addition, polymer surface morphology and particle size distribution were determined.

### 2.6. Dye Degradation by a Pulsed Light Advanced Oxidation Process

A standardized method [17], was followed. In brief, the method uses a mixture of dye and hydrogen peroxide, which is subjected to light pulses of broad-spectrum high-intensity light by means of a XeMaticA-Basic-1L system (Steribeam, Germany). In the current case, the following conditions were used: Sample volume: 20 mL, 45 mg/L dye, 3000 mg/L H_2_O_2_ and 70 pulses (150 J/cm^2^).

### 2.7. Supplementary Information

The quantity of solute adsorbed on the EPI-cross-linked CDs (*q_t_*), in mg/g, was determined according to Renard et al. (1997) [37], using the Equation (1):

(1) $$q_{e}=\frac{V(C_{o}-C_{e})}{m},$$

where *C_o_* is the initial concentration of dye in the liquid phase (mg/L), *C_e_* the liquid phase dye concentration at equilibrium (mg/L), *V* the volume of dye solution employed (L) and *m* is the mass of polymer utilized (g).

The adsorption kinetics allows predicting the rate of dye removal and offers data for determining the sorption mechanism of the dye into the polymers, and could be evaluated by the following equations:

The pseudo first order rate of Lagergren (1898) [38], given by the Equation (2):

(2) $$\ln\left(q_{e}-q_{t}\right)=\ln q_{e}-k_{1}t,$$

where *q_e_* and *q_t_* are the concentrations of dye adsorbed (mg/g) at equilibrium, and at time t (min) respectively, and *k*_1_ (min^−1^) is the pseudo first order rate constant. Values of *k*_1_ were worked out from the plot of ln (*q_e_* − *q_t_*) versus *t*.

The pseudo second order model [39] was evaluated using the Equation (3):

(3) $$\frac{t}{q_{t}}=\frac{1}{{k_{2}q_{e}}^{2}}+\frac{1}{q_{e}}t,$$

where *q_e_* symbolize the concentration of dye absorbed at equilibrium (mg/g), *q_t_* the amount of dye absorbed at time *t* (mg/g) and *k*_2_ is the equilibrium rate constant of pseudo second order.

Also, by the Elovich model, this is presented by the following Equation (4), [40]:

(4) $$q_{t}=\frac{1}{\beta }\ln\alpha \beta +\frac{1}{\beta }\ln t,$$

where α symbolize the initial adsorption rate (mg/g min), and β is the desorption constant (g/mg).

The intraparticle diffusion model [41], where the root time dependence can be determined by the following Equation (5):

(5) $$q_{t}=k_{i}\sqrt{t}+C,$$

where *q_t_* is the quantity of solute on the surface of the sorbent at time *t* (mg/g), *k_i_* the intraparticle diffusion rate constant (mg/g min^1/2^), *t* the time and *C* is the intercept (mg/g). The *k_i_* values are obtained from the slopes of *q_t_* versus *t*^1/2^ plots.

In addition, for the determination of the distribution of dye molecules between the liquid and the solid phase, Freundlich, Langmuir and Temkin adsorption isotherms were fitted to the experimental data using the following equations:

The linearized form of the Freundlich model is given by the following Equation (6):

(6) $$\ln q_{e}=\ln K_{F}+\frac{1}{n_{F}}\ln C_{e},$$

where *q_e_* is the equilibrium adsorption capacity (mg/g), *C_e_* the equilibrium dye amount in solution (mg/L), *K_F_* the Freundlich constant (L/g) and 1/*n_F_* is the heterogeneity factor. The plot of ln *q_e_* versus ln *C_e_* was used to obtain the intercept value of *K_F_* and the slope of 1/*n_F_*. The linearized form of the Langmuir model is given by the following Equation (7):

(7) $$\frac{C_{e}}{q_{e}}=\frac{1}{K_{L}}+\frac{a_{L}}{K_{L}}C_{e},$$

where *C_e_* (mg/L) and *q_e_* (mg/g) are the liquid phase concentration and solid phase concentration of adsorbate at equilibrium respectively. *K_L_* (L/g) and *a_L_* (L/mg) are the Langmuir isotherm constants. Plotting *C_e_*/*q_e_* versus *C_e_* is possible to know the value of *K_L_* from the intercept (1/*K_L_*) and the value of *a_L_* from the slope (*a_L_*/*K_L_*); *q_max_* is the maximum adsorption capacity of the polymer and is defined by *K_L_*/*a_L_*.

The linearized form of the Temkin model is given by the following Equation (8):

(8) $$q_{e}=\frac{R_{T}}{b_{T}}\ln a_{T}+\frac{R_{T}}{b_{T}}\ln C_{e},$$

where *b_T_* is the Temkin constant, related to the heat of adsorption (kJ/mol), *a_T_* is the constant of Temkin isotherm (L/g), *R* is the universal gas constant (8.314 J/mol∙K) and *T*, the absolute temperature in Kelvin.

## 3. Results and Discussion

The first step in the adsorption procedure is to select the most desirable conditions to achieve the maximum removing of dye. Different parameters should be taken into account, some of them are: Quantity of adsorbent, effect of pH, agitation speed, temperature or dye concentration. After considering all these aspects, the experimental results indicated us that 1 g of polymer, pH 7.0, 500 rpm of agitation speed were the best conditions to carry out the rest of experiments (data not shown). Since adsorption is usually influenced by temperature, experiments were carried out at 25, 40 and 50 °C, selecting 25 °C as optimal since it led to higher *q_e_* values (data not shown). These conditions were previously tested by our research group, when the adsorption of Direct Red 83:1 using α and HP-α-CDs-EPI was carried out [36]. First, a detailed kinetic study was developed to understand the adsorption process, followed by the analysis of the different isotherm models to know which isotherm presented the best fit to the experimental data.

### 3.1. Effect of Contact Time

**Figure 1:**
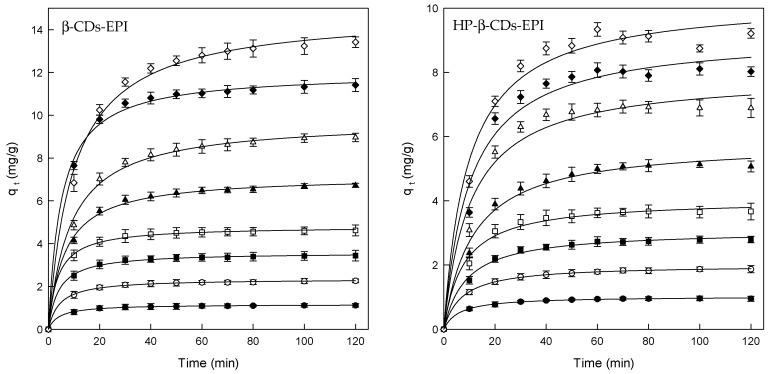
Amount of Direct Red 83:1 adsorbed by β-CDs-EPI and HP-β-CDs-EPI as function of contact time, for different dye concentrations. 25 mg/L (●), 50 mg/L (○), 75 mg/L (■), 100 mg/L (□), 150 mg/L (▲), 200 mg/L (Δ), 250 mg/L (♦) and 300 mg/L (⟡).

The effect of contact time on the adsorption of Direct Red 83:1, and CDs-EPI polymers was determined using different concentrations of dye and the standard conditions mentioned above. The results of these experiments could be observed in Figure 1.

The adsorption rate rapidly increased in the first 20 min of contact, remaining constant until the end of the process. The faster adsorption is attributed to the great number of the adsorbent active binding sites, and with progressive occupancy of these sites, the adsorption becomes less efficient in the final stages [42].

Despite the fact that the equilibrium time was found to be 20 min approximately, the contact time was fixed at 120 min for the rest of the experiments to ensure that equilibrium was completely achieved. 

Table 1 shows the effect of the initial Direct Red 83:1 concentration on the adsorption of dye by the CDs-EPI polymers. When the initial amount of Direct Red rose from 25 to 300 mg/L, the adsorption capability increased from 1.1 to 13.4 and from 0.9 to 9.3 mg/g for β- and HP-β-CDs-EPI, respectively.

The higher concentration of dye increases the adsorption into the polymers because the probability of collision between both molecules is higher, and there is a larger concentration gradient between the liquid and CD cavities that allows a stronger driving force to overcome all mass transfer resistances between the aqueous and solid phases [43].

Therefore, the increase in the initial Direct Red 83:1 concentration enhanced the adsorption of the dye by the CDs-EPI polymers.

### 3.2. Adsorption kinetics

**Figure 2:**
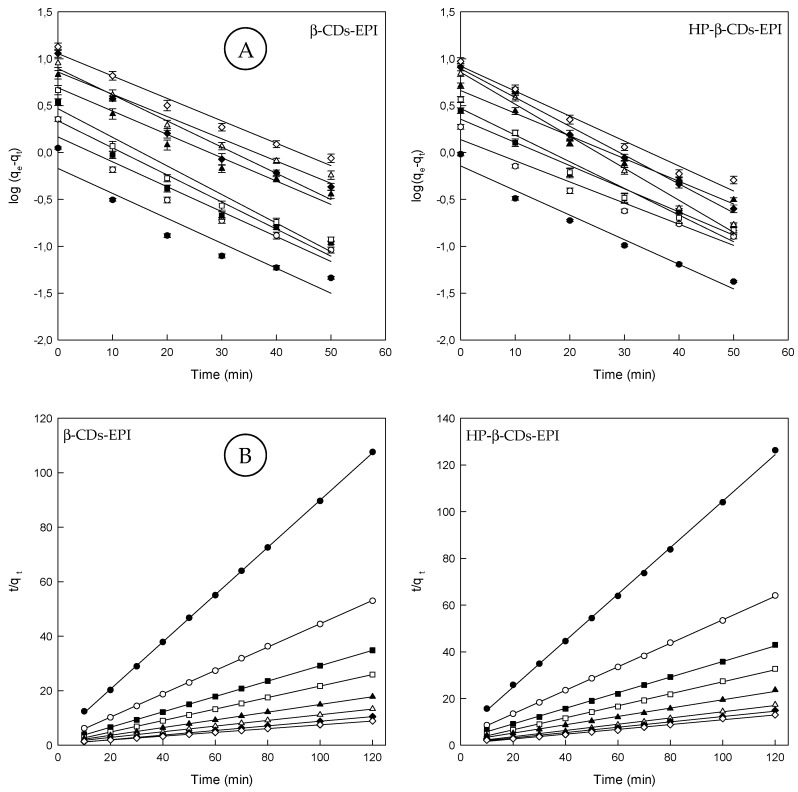
(**A**) Pseudo first order model plots and (**B**) pseudo second order model plots for the Direct Red 83:1 adsorption onto β-CDs-EPI and HP-β-CDs-EPI polymers at different dye concentrations (25 mg/L (●), 50 mg/L (○), 75 mg/L (■), 100 mg/L (□), 150 mg/L (▲), 200 mg/L (Δ), 250 mg/L (♦) and 300 mg/L (⟡)).

The kinetics of Direct Red 83:1 adsorption on both adsorbents was studied in order to establish the time of equilibration for the maximum adsorption, and the rate determining step of the adsorption. Thus, pseudo first order, pseudo second order, the intraparticle diffusion model, the diffusion model and Elovich model were employed in this study. Figure 2A, showed the results obtained for both polymers when the pseudo first order rate was applied to the experimental data (see Section 2.7). The calculated parameters for this model are shown in Table 2. One of the main drawbacks of this model is the impossibility to obtain straight lines when all the contact times are considered.

As could be noticed in Figure 2A, only the first 50 min of contact between the dye and polymers were considered in an attempt to obtain these straight lines. This reduction allowed us to obtain *R*^2^ values higher than 0.9 in all cases analyzed.

Apart from that, the experimental (*q*_eexp_) and calculated (*q*_ecal_) values estimated from the pseudo first order model were found different, indicating that this was not an appropriate model to explain the kinetic behavior of the polymers. This fact suggests that the model is adequate only for the first stages of adsorption, since the adsorption is fast. Therefore, a new adjustment was carried out using a pseudo second order model (see Section 2.7). Figure 2B shows the pseudo second order model with the experimental data and the respective kinetic parameters are shown in Table 2.

Comparing the pseudo first and pseudo second order models, it could be stated that the linear determination coefficient is lower in all cases, when the pseudo first order model was used. The values of *R*^2^ for the pseudo second order model are clearly higher (>0.99) and the determined values (*q*_ecal_) were closer to the experimental ones (*q*_eexp_) (Table 2).

Both parameters indicated the great adjustment of this model to the experimental data obtained. 

Also, from Table 2, the values of rate constant (*k*_2_) decreased with initial dye concentration. At higher concentrations, the competition for polymer surface active sites will be high, and consequently lower sorption rates could be achieved [44].

**Figure 3:**
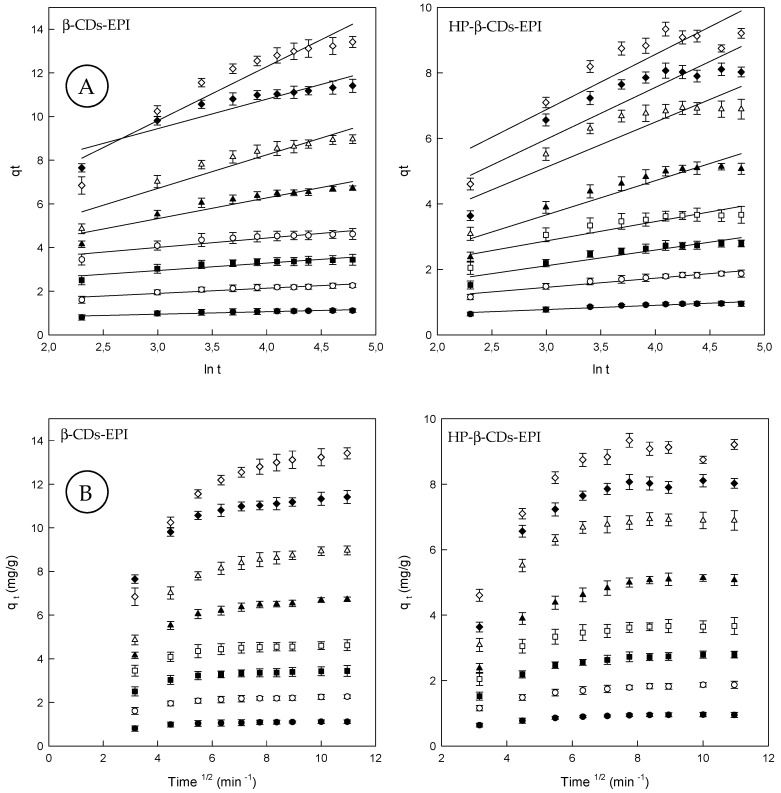
(**A**) Elovich model plots for the Direct Red 83:1 adsorption onto β-CDs-EPI and HP-β-CDs-EPI polymers. (**B**) Intraparticle diffusion model plots for the Direct Red 83:1 adsorption onto β-CDs-EPI and HP-β-CDs-EPI polymers at different dye concentrations (25 mg/L (●), 50 mg/L (○), 75 mg/L (■), 100 mg/L (□), 150 mg/L (▲), 200 mg/L (Δ), 250 mg/L (♦) and 300 mg/L (⟡)).

Although the Elovich model does not predict any definite mechanism, could be useful in describing predominantly chemical adsorption on heterogeneous adsorbents (see Section 2.7). In fact, Teng and Hsieh (1999) [45], proposed that constant α is related to the rate of chemisorption, while β is correlated to the surface coverage. The constants depend on the amount of adsorbent with the adsorption constant, α, being the most sensitive parameter. The plot of *q_t_* against ln*t* provides a linear relationship, which *α* and *β* are determined from the slop and intercept of the plot (Figure 3A).

Since α characterizes the initial adsorption rate, the results reveal that the adsorption rate can be improved by increasing the concentration of dye for both polymers (Table 2). A higher value of α indicates that a better adsorption mechanism is observed [46]. According to the *R*^2^ values observed for the Elovich model, it can be concluded that the pseudo second order model presented the best fit to the experimental values suggesting that chemisorption is the rate-controlling step.

Finally, by means of the intraparticle diffusion model, the kinetic results were evaluated to elucidate the adsorption behavior of Direct Red 83:1 on CDs-EPI polymers (see Section 2.7). This model is noteworthy to recognize the rate-limiting step in the adsorption process. This step can be either the boundary layer (film), or the intraparticle diffusion (pore) of solute on the solid surface from the bulk of the solution in a batch process. In diffusion studies, the rate is expressed in terms of the square root time. Figure 3B, showed that the adsorption plots are not linear over the entire time range, and can be separated into two linear regions, which confirm the multi stages of adsorption.

The first step involves external mass transfer followed by intraparticle diffusion. This means that the dye molecules were carried to the external surface of the CDs-EPI polymers by means film diffusion, and its rate was very fast. Afterward, dye molecules were entered inside the polymers by intra particle diffusion. This fact displayed that the intra particle model is involved in the adsorption process, but not the only rate-controlling step. When the lines do not intercept the origin, it indicates that film diffusion and intra particle diffusion happened simultaneously [47]. The intraparticle diffusion rate constant (*k_i_*), which is the slope of the lineal zone, is listed in Table 2, whereas the intercept of each curve, is proportional to the boundary layer thickness. In general, *k_i_* values become higher with increasing concentrations of Direct Red 83:1. The *R*^2^ values were comprised between 0.6 and 0.8. The C (*q_e_*) values found supply information about the thickness of the boundary layer; a higher intercept suggests a bigger effect of it. This value increased with increasing dye concentrations (Table 2).

### 3.3. Adsorption Equilibrium

**Figure 4:**
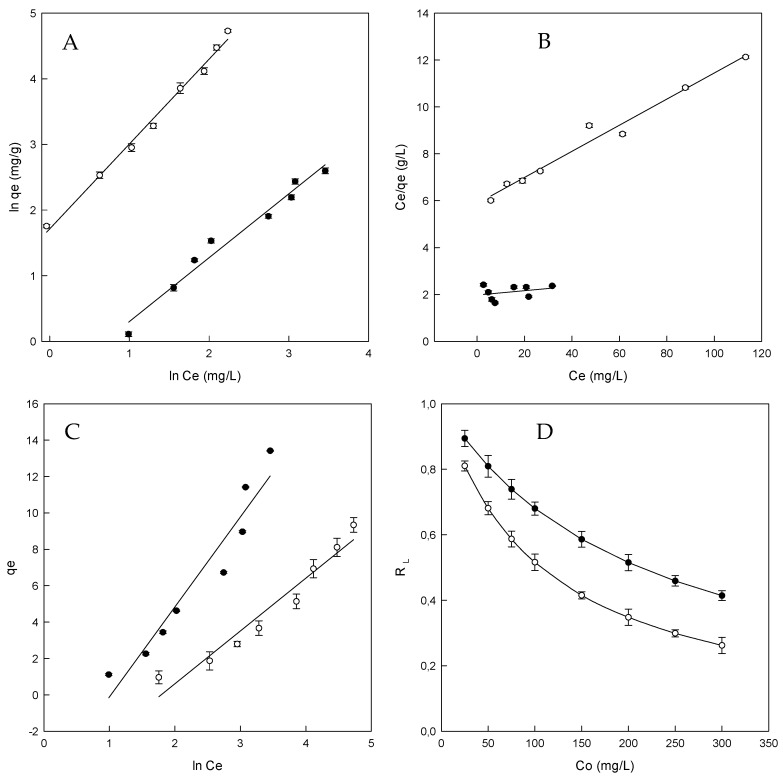
Adsorption isotherms for Direct Red 83:1 by β-CDs-EPI (●) and HP-β-CDs-EPI (○). (**A**) Freundlich isotherm, (**B**) Langmuir isotherm, (**C**) Temkin isotherm, (**D**) Separation factor.

The adsorption isotherm is essential to describe how the dye will interact with the adsorbent and gives an approach to the adsorption capacity of the adsorbent [48]. Freundlich isotherm model is used for adsorption on heterogeneous surfaces and multilayer sorption. The application of this model also indicates that the energy decreases on completion of the sorptional centers of an absorbent Weber and Morris (1962) [41]. The results for the Freundlich model (see Section 2.7), are given in Figure 4A and Table 3. 

The adjustment of the model is appropriate for the adsorption system under the range of concentrations employed (*R*^2^ 0.971 and 0.991 for β- and HP-β-CDs-EPI, respectively). The value of *R*^2^ is higher than the other two isotherms and the value of *n_F_* was higher than 1.0 in both cases (1.028 for β-CDs-EPI and 1.305 for HP-β-CDs-EPI), which indicates the favorable adsorption process.

Due to these facts, it can be concluded that the Freundlich equation, supplies a rational description of the experimental data [49].

Langmuir isotherm model considers that the adsorption process is developed at homogeneous sites on the polymer and is widely used for the adsorption of a pollutant from a liquid solution [50]. The Langmuir equation is appropriate for homogeneous sorption (see Section 2.7), where the sorption of each molecule onto the surface possess same sorption activation energy.

A dimensionless constant (*R_L_*) which is called separation factor, described the most relevant characteristic of this isotherm, depicted by Equation (9):

(9) $$R_{L}=\frac{1}{1+a_{L}C_{o}},$$

where *C_o_* is the initial dye amount (mg/L) and *a_L_* is the Langmuir constant (L/mg). The adsorption process is indicated by the value of *R_L_*, being *R_L_* > 1 an unfavorable process, linear for *R_L_* = 1, favorable for *R_L_* between 0 and 1 or irreversible for *R_L_* = 0.

The plot of Langmuir isotherm is given in Figure 4B and the parameters obtained are gathered in Table 3. The value of *q_max_* is very interesting because the maximum adsorption capacity for β-CDs-EPI (107.5 mg/g) was six times higher than in the case of HP-β-CDs-EPI (18.2 mg/g).

The results obtained for β-CDs-EPI (0.894–0.414) and HP-β-CDs-EPI (0.810–0.262) were between 0 and 1, indicating that the adsorption process was favorable (Figure 4D and Table 3).

Regarding the *R*^2^ values for the Langmuir isotherm (0.710 and 0.976 for β- and HP-β-CDs-EPI respectively), it can be stated that this model did not fit well. This value is lower than the Freundlich isotherm value. When the Freundlich isotherm yields a better fit than the Langmuir isotherm, it could be suggested that the boundary layer thickness is increased [51].

On the contrary that describes the equation of Freundlich, Temkin isotherm assumes that the fall in the heat of sorption is linear rather than logarithmic. The Temkin equation (see Section 2.7), indicates a linear decrease of sorption energy as the level of completion of the sorptional centres of an adsorbent is increased [50].

From the Temkin isotherms (Figure 4C), typical bonding energy range for the ion exchange mechanism was reported to be in the range of 8–16 kJ/mol, while the physic sorption process was reported to have adsorption energies less than −40 kJ/mol [52].

The *b_T_* value calculated for β-CDs-EPI and HP-β-CDs-EPI were 0.5 kJ/mol and 0.85 kJ/mol, respectively. These results suggested that in the adsorption of Direct Red 83:1 by the CDs polymers were involved in physical and chemical processes. The determination coefficient is lower than Freundlich and Langmuir values. For that reason, it is clear that the Freundlich model yields a much better fit than the Temkin model.

The standard free energy (Δ*G*^0^) of the adsorption process was obtained at 25 °C using Equation (10):

Δ*G*^0^ = −RT ln *K_L_* (10)

where *T* is the temperature of the solution in Kelvin, *R* the universal gas constant (8.314 J/mol K) and *K_L_* is the Langmuir constant. The Δ*G*^0^ values were 1.7 kJ/mol and 4.4 kJ/mol for β-CDs-EPI, and HP-β-CDs-EPI, respectively.

Positive Δ*G*^0^ values were obtained at 25 °C, demonstrating that spontaneity was not favoured at low temperatures. A similar tendency was observed in the adsorption of Direct Red 83:1 using EPI polymers made of α- and HP-α-CDs [36].

The comparison of the adsorption capacities of dissimilar polymers made of CDs with different dyes is always interesting to evaluate the properties and effectiveness of the new EPI polymers developed. To clarify the situation, the value of *q_max_* is appropriate to make comparisons among different adsorbents. Table 4 presents recent applications of CDs cross-linked with EPI or other cross-linking agents as dye adsorbent materials for water treatment.

According to the adsorption indices presented in Table 4, the β-CDs-EPI polymer could be used as effective and promising sorbent in liquid-solid sorption procedures for removal of azo dyes. These polymeric adsorbents have proved to be more efficient and advantageous over conventional systems. In fact, epichlorohydrin polymers are easy to synthesize, their chemistry is based on aqueous systems in a single step.

Also, they are effective in the removal of contaminants (including traces), since exhibit high sorption capacities and relative rapid kinetics, being able to reuse them in successive sorption processes since can be regenerated using ethanol or acetate buffer as washing solvents, without appreciable loss of capacity of sorption, contrasting active carbons and more easily than resins [32]. However, these materials present sorption mechanisms that are still being elucidated since entail various interactions that could arise simultaneously, complicating the possible explanation of the results [60].

Even though there are many reports demonstrating the existence of a mechanism of chemisorption through the inclusion complexes formation (besides stabilizing forces), another mechanism is becoming broadly acknowledged in recent years. Different studies have emphasized the role performed by the macromolecular network formed through a cross-linking agent. To explain this behavior, has been introduced the concept of association complex that evidence both the role of the CD molecules and of the 3D macromolecular network of the materials in the overall sorption mechanism [32].

### 3.4. Morphology

**Figure 5:**
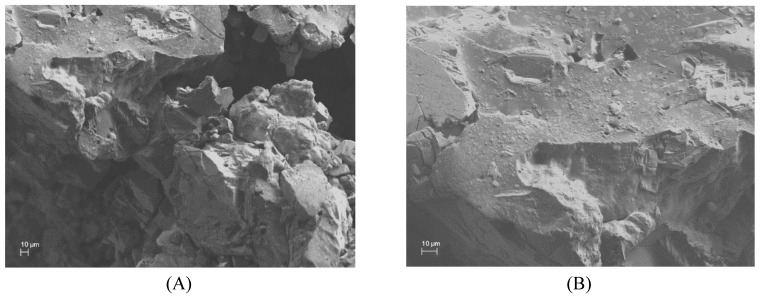
SEM images of cross-linked β-CD-EPI empty polymer (**A**) and laden with Direct Red 83:1 (**B**).

The shapes of the cross-linked adsorbents were investigated by SEM. As shown in (Figure 5A), β-CDs-EPI polymers were distinguished for its porous and irregular structure, suitable to entrap the dye molecules. This process forced a smoother morphology of the polymer surface (Figure 5B), suggesting that Direct Red 83:1 was accumulated over its surface.

The particle size distribution, expressed as the mean volumetric size D [4:3] was higher for the native β-CDs (76 µm) with respect to the modified HP-β-CDs (31 µm), similar results to those obtained after polymerization step, 586 µm and 516 µm for β-CDs-EPI and HP-β-CDs-EPI, respectively.

The particle size distribution was estimated by span values, which were 2.8; 1.3; 5.8 and 1.7 for β-, HP-β-, β-CDs-EPI and HP-β-CDs-EPI, respectively, showing better distribution HP-β-CDs-EPI polymer, since a more homogeneous distribution was evidenced for lower values.

**Figure 6:**
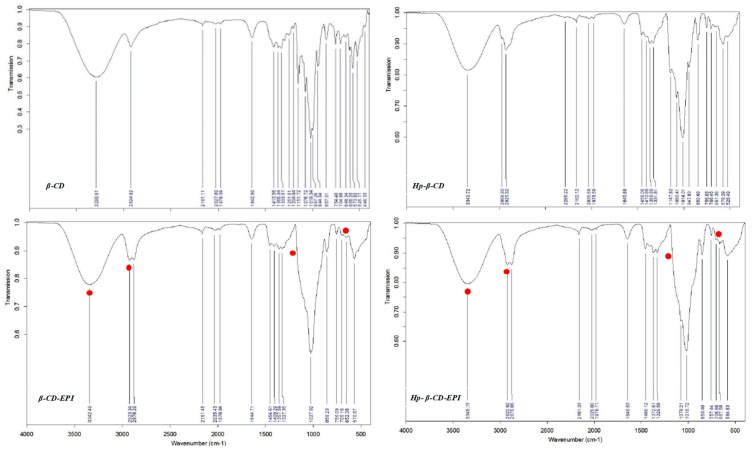
β-CD, HP-β CD, β- and HP-β-CD EPI polymers FT-IR spectra.

FTIR was used to determine the polymers surface functional groups, responsible for interactions with the dye. Even though β- and HP-β CDs spectra show very similar structure (Figure 6), since present analogous functional groups, it was possible to recognize little variances provoked by the presence of methyl groups in HP-β CDs.

Table 5 summarizes the FT-IR characteristic bands of the IR spectrum of β- and HP-β-CD monomers.

The IR spectrum of β-, HP-β-CD-EPI polymers reveal CH_2_Cl wagging bands (1286.27, 1257.33), and C–Cl stretching at 756.09 and 757.44 cm^−1^, respectively (Figure 6, filled red points), corresponding to epichlorohydrin crosslinker typical peaks. 

The stretching vibrations of OH, CH_2_ and C–O–C are around 3342.48, 2923.34, 2878.29, and 1083.79 for β-CD-EPI, and 3348.23, 2922.92, 2876.86 and 1089.57 for HP-β-CD-EPI, respectively; verify the presence of cyclodextrin in the structure.

**Figure 7:**
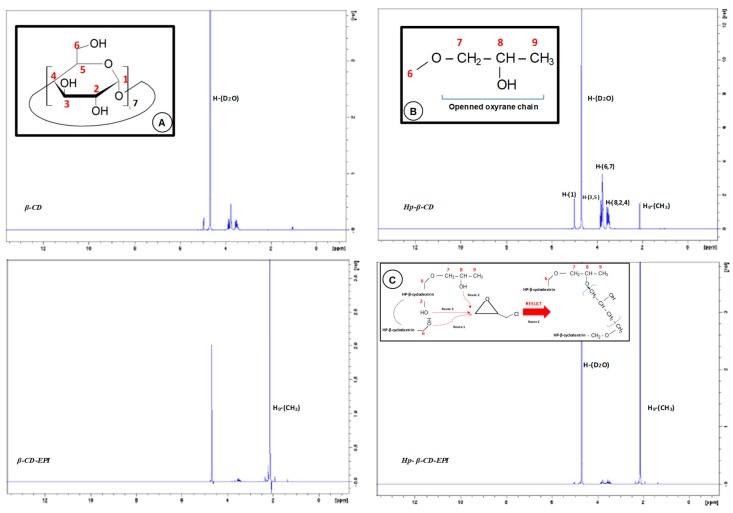
^1^H-NMR spectra of β-CD, HP-β CD, β- and HP-β-CD EPI polymers. Inset: A: β-CD monomer glucose unit chemical structure; B: HP-β-CD spacer arm; C: Possible ways to open oxyrane ring by OH groups.

The ^1^H-NMR spectrum of β-CD exhibit two kinds of signals (Figure 7): One peak close to 5 ppm corresponding to the anomeric proton linked to the C-(1) of the glucose unit, and two widen peaks between 3.3 and 4 ppm related to the hydrogens of the pyranose rings. 

Throughout β-CD polymerization reaction, epoxide ring breaks to give the glycerol unit and protons of this unit resonates in the 3.2-3.8 ppm interval, producing the chemical rearrangement a significant modification in the spectrum signals. The signal ascribed to the hydrogen atoms of the 2-hydroxypropyl ether segment resonance [C-(7-8)], is displaced below the two peaks of the pyranose units. As a result, the increment of five hydrogen atoms by one EPI molecule is shown in the integration value of these peaks. 

A single peak close to 2.1 ppm agree with –CH_3_ protons (H_9_) of terminal methyl of the hydroxypropyl radical, that does not appear in the spectrum of the β-CD, that is more intense for EPI polymers. A single peak observed in all spectra at 4.6 ppm, is due to D_2_O solvent. Since as the degree of substitution in the alkyl moiety diminish the acidity of the alcohols decreases, the OH of C_6_ of glucopyranose unit (primary alcohol) will be more reactive than secondary ones located at C_8_ in HP-β-CD, arranging hence two routes of attack to the EPI molecule, depending on the number on hydroxypropyl R groups. Also, could be possible another more limited route (route 3), due to the ring strain. When either the polymer is modified, or both the crosslinking and modification steps are simultaneously accomplished, the hydroxyl groups on the glyceryl bridges and on glyceryl monoether polymer side chains are reactive as well. Therefore, the reactive alkoxy groups can be sited both on the CD rims and on the network. Contrary to what happened with the β-CD, in this case the chemical modification gives no significant change in the spectrum signals. 

**Figure 8:**
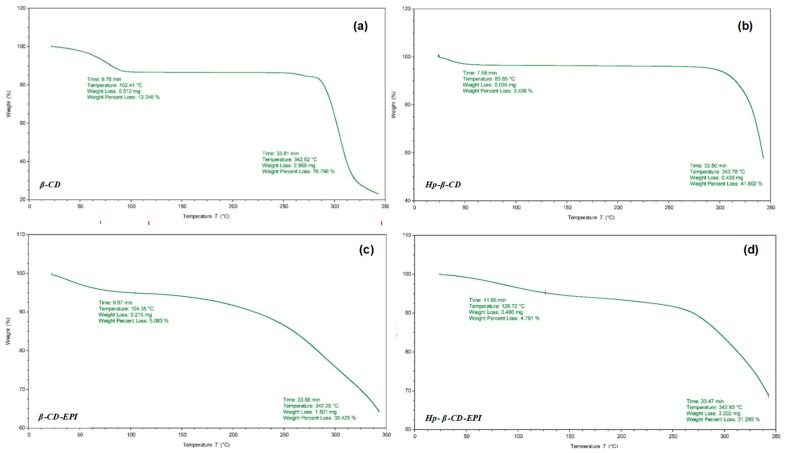
Thermogravimetric curves for the samples of (**a**) β-CD, (**b**) HP-β-CD, (**c**) β-CD-EPI, and (**d**) HP-β-CD-EPI polymers.

In addition, the TG curves for β-, HP-β-CDs and their polymers were carried out. The TG curve for β-, HP-β-CDs (Figure 8A,B) revealed three mass loss zones. 

Firstly, between 30 and 102.4 °C for β-CDs and 83.85 °C for HP-β-CDs, was due to dehydration and accounted for around 13.25% and 3.45, respectively, of the mass reduction. The second, between 290 and 342 °C (exothermic) was due to decomposition of the organic groups, with a mass loss of 76.8% and 41.8%, respectively. The respective CD-EPI polymers (Figure 8C,D) also presented three phases of weight loss, but the curves show a similar trend and a softer descent than non-polymerized CDs, as well as lower mass losses in the exothermic section (290 and 342 °C), which were 36.4% for β-CD-EPI and 31.28% for HP-β-CD-EPI polymers.

The third phase, among 350 and 560 °C, was due in all cases, to degradation of the final residues. These results indicate that both polymers present a similar TGA behavior, as well as their stability was significantly improved after polymerization process.

### 3.5. Dye Degradation by a Pulsed Light Advanced Oxidation Process

**Figure 9:**
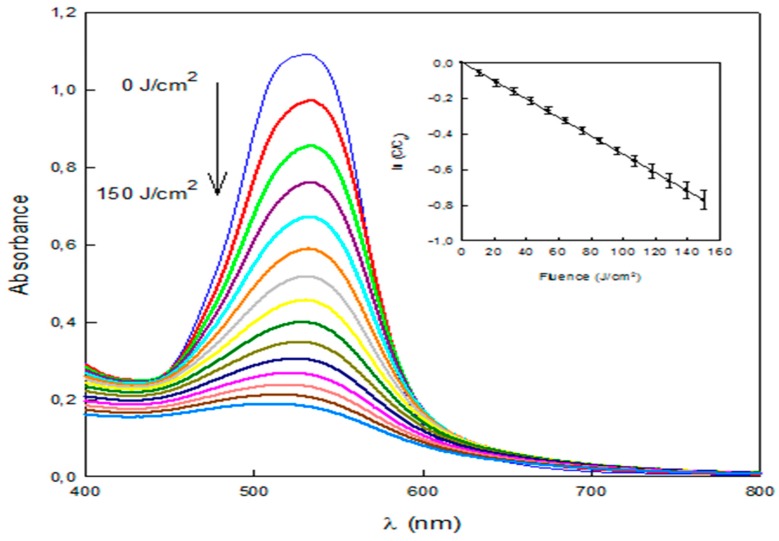
Spectral changes during the course of the degradation of Direct Red 83:1 by a pulsed light/H_2_O_2_ advanced oxidation process. Inset: Pseudo-first order kinetic plot.

Cyclodextrin polymers do not adsorb all the amount of dyes present in water. Therefore, a complementary process was tested in order to further reduce the quantity of dye that would be released into the environment. To this end, dye degradation by an innovative AOP where the light source is a pulsed light system was tested [17]. The process was able to degrade Direct Red 83:1 efficiently, as it can be observed in the flattening of the visible light spectrum (Figure 9). The degradation followed a pseudo-first order kinetics (Figure 9, inset) (*R*^2^ = 0.9998) with a decolorization constant of 0.0052 cm^2^/J. The concentration of Direct Red 83:1 in water was reduced by >80% by this process with the application of 150 J/cm^2^. Furthermore, the degradation kinetics let think that nearly full degradation is achievable by extending the treatment time.

Since the β-CDs-EPI polymer was able to remove 89.7% of dye from wastewater, the subsequent application of the AOP would leave only <2% of dye in water allowing reusing the treated wastewater, appearing cleaner because the dye that was not entrapped with the polymer was destroyed.

Furthermore, it was demonstrated (data not shown) that the proposed methodology has not only the capability to eliminate dyes from wastewater, diminishing more than 5% the water pollution, but also the ability to desorbs them for further dyeing processes using acetate buffer pH 3. The polymer works well up to six adsorption/desorption cycles without performance and structure losses. Considering the point of view of a sustainable recycling economy in the textile dyeing process, the proposed methodology is environmental and economically advantageous for any dyeing enterprise, such as Colorprint, since for dyeing 1 kg of textile the cost of dye in wastewater is around 0.092 €, with dye waste annual costs of 25.000 €/year.

## 4. Conclusions

The synthesis of insoluble polymers using β-CDs and HP-β-CDs oligosaccharides as starting material and EPI as cross-linking agent was proved to be thoroughly viable, yielding a material that could be used as effective and promising sorbent in liquid-solid sorption procedures for azo dyes removal from wastewater effluents. 

The β-CDs-EPI polymer presented a high adsorption capacity for Direct Red 83:1 (*q_max_* = 107.5 mg/g), almost six times higher than HP-β-CDs-EPI (*q_max_* = 18.2 mg/g), which compares favorably with other commonly used and more expensive adsorbents. 

The polymer synthesis was confirmed by FTIR, which evidenced alterations in the vibrational modes, as well as CH_2_Cl wagging and C–Cl stretching bands which are distinctive peaks for epichlorohydrin crosslinker. 

The ^1^H-NMR spectrum evidenced that the polymers obtained showed a single more intense peak, corresponding to (H9) that is weaker for HP-β-CDs and does not appear in the spectrum of the β-CD. 

Thermogravimetric analyses showed differences between the degradation temperatures of the precursors and its respective polymers, showing these lower mass losses (40% β-CD-EPI and 10% HP-β-CD-EPI) in the exothermic section.

The results indicated that the pseudo second order model presented the best fit to the experimental data, indicating that chemisorption is controlling the process. The equilibrium experimental data were satisfactorily fitted using the Freunlich model, which indicates that the adsorption process is happening on heterogeneous surfaces, involving physical and chemical processes.

The results obtained show that β-CDs-EPI polymer could be used for removal of Direct Red 83:1 as a promising alternative over other more expensive adsorbents. Its advantages include good adsorption properties, ease of preparation and relatively low cost. 

This polymer could contribute to diminishing environmental impacts caused by the arbitrary discharge of dye effluents into aquatic systems, while reducing the costs of treatment of wastes destined to be either discharged or reused in production processes. 

A pulsed light advanced oxidation process can efficiently degrade those amounts of dye that are not retained by CDs in order to minimize the environmental impact of these residues.

## Figures and Tables

**Table 1:** Effect of the initial dye concentration on the adsorption capacity of the polymers.

		Direct Red 83:1 Concentrations (mg/L)							
***q_e_* (mg/g)**	**Polymer**	25	50	75	100	150	200	250	300
	**β-**	1.1	2.3	3.5	4.6	6.7	9.0	11.4	13.4
	**HP-β-**	0.9	1.9	2.8	3.7	5.1	6.9	8.1	9.3

**Table 2:** Kinetics parameters for the Pseudo First and Second Order; Elovich Mass Transfer and Intraparticle Diffusion Models.

**PFOM**	**β-CDs-EPI**				**HP-β-CDs-EPI**			
***C_o_* (mg/L)**	***q*_eexp_**	***q*_ecal_**	***k*_1_ (min^−1^)**	***R*^2^**	***q*_eexp_**	***q*_ecal_**	***k*_1_ (min^−1^)**	***R*^2^**
**25**	1.115	0.676	0.061	0.904	0.961	0.719	0.060	0.970
**50**	2.263	1.465	0.061	0.934	1.871	1.367	0.051	0.950
**75**	3.442	2.167	0.066	0.930	2.793	2.259	0.057	0.969
**100**	4.621	2.924	0.070	0.943	3.668	2.944	0.065	0.967
**150**	6.723	4.897	0.057	0.948	5.136	4.570	0.056	0.985
**200**	8.964	7.211	0.055	0.968	6.934	6.309	0.078	0.990
**250**	11.413	7.852	0.064	0.944	8.110	7.834	0.070	0.991
**300**	13.415	11.324	0.055	0.978	9.338	8.336	0.061	0.977
**PSOM**	**β-CDs-EPI**				**HP-β-CDs-EPI**			
***C_o_* (mg/L)**	***q*_eexp_**	***q*_ecal_**	***K*_2_ (min^−1^)**	***R*^2^**	***q*_eexp_**	***q*_ecal_**	***K*_2_ (min^−1^)**	***R*^2^**
**25**	1.115	1.153	0.232	0.999	0.961	1.0	0.2	0.999
**50**	2.263	2.336	0.104	0.999	1.871	1.988	0.074	0.999
**75**	3.442	3.546	0.0821	0.999	2.793	2.994	0.045	0.999
**100**	4.621	4.761	0.0681	0.999	3.668	3.906	0.044	0.998
**150**	6.723	7.092	0.0243	0.999	5.136	5.617	0.0193	0.996
**200**	8.964	9.615	0.0133	0.999	6.934	7.518	0.0176	0.993
**250**	11.413	11.834	0.0197	0.999	8.110	8.771	0.0149	0.993
**300**	13.415	14.471	0.00821	0.998	9.338	9.803	0.0154	0.993
**EMTM**	**β-CDs-EPI**				**HP-β-CDs-EPI**			
***C_o_* (mg/L)**		**α**	**β**	***R*^2^**		**α**	**β**	***R*^2^**
**25**		1.831	8.771	0.868		1.472	7.751	0.904
**50**		3.254	4.166	0.885		1.845	3.571	0.934
**75**		6.841	2.932	0.853		1.948	2.083	0.880
**100**		15.119	2.325	0.855		2.992	1.692	0.817
**150**		11.834	1.052	0.881		1.698	0.956	0.871
**200**		8.076	0.649	0.894		2.669	0.725	0.772
**250**		22.08	0.740	0.844		1.472	7.751	0.904
**300**		11.02	0.404	0.888		1.845	3.571	0.934
**IDM**	**β-CDs-EPI**				**HP-β-CDs-EPI**			
***C_o_* (mg/L)**	***q*_eexp_**	***q*_ecal_**	***K_i_* (mg/g min^1/2^)**	***R*^2^**	***q*_eexp_**	***q*_ecal_**	***K_i_* (mg/g min^1/2^)**	***R*^2^**
**25**	1.115	0.807	0.0329	0.730	0.961	0.613	0.037	0.770
**50**	2.263	1.600	0.0693	0.751	1.871	1.094	0.082	0.816
**75**	3.442	2.529	0.0974	0.708	2.793	1.513	0.138	0.740
**100**	4.621	3.481	0.122	0.708	3.668	2.160	0.166	0.661
**150**	6.723	4.139	0.273	0.745	5.136	2.379	0.298	0.726
**200**	8.964	4.779	0.444	0.760	6.934	3.506	0.382	0.606
**250**	11.413	7.796	0.384	0.698	8.110	4.111	0.440	0.615
**300**	13.415	6.735	0.711	0.750	9.338	4.879	0.471	0.633

PFOM: Pseudo first order model; PSOM: Pseudo second order model; EMTM: Elovich Mass Transfer Model; IDM: Intraparticle diffusion model.

**Table 3:** Adsorption isotherm constants obtained for β- and HP-β-CDs EPI polymers.

Isotherm	Parameter	β-CDs-EPI	HP-β-CDs-EPI
**Freundlich**	*K_F_*	0.511	0.272
	*n_F_*	1.028	1.305
	*R* ^2^	0.971	0.991
**Langmuir**	*q_max_*	107.5	18.2
	*K_L_*	0.506	0.170
	*a_L_*	0.0047	0.0094
	Δ*G*	1.68	4.39
	*R* ^2^	0.705	0.976
**Temkin**	*R_L_*	0.894–0.414	0.810–0.262
	*a_T_*	0.359	0.167
	*b_T_*	0.50	0.85
	*R* ^2^	0.929	0.945

**Table 4:** Dye adsorption capacity of different CDs polymers.

Dye	Polymer	*q_max_* (mg/g)	Reference
Methylene Blue	β-CDs-AH	333	[53]
Rhodamine B	β-CDs-AH	250	[53]
Direct Red 83:1	β-CDs-EPI	107.5	This work
Methylene Blue	β-CDs-CA	105	[54]
Malachite Green	β-CDs-EPI-CMC	91.9	[55]
Basic Blue 9	β-CDs-EPI-CMC	56.5	[51]
Congo Red	β-CDs-HMDI	36.2	[56]
Direct Red 83:1	α-CDs-EPI	31.5	[36]
Direct Red 83:1	HP-α-CDs-EPI	23.4	[36]
Direct Red 83:1	HP-β-CDs-EPI	18.2	This work
Brilliant Yellow	HP-β-CDs-CS	8.8	[57]
Methylene Blue	β-CDs-SI	187.6	[58]
Methylene Blue	CM-β-CD-MNP(C)	140.8	[59]
Methylene Blue	CM-β-CD-MNP(P)	277.8	[59]

AH: Anhydride, EPI: Epichlorohydrin, CA: Citric acid, EPI-CMC: Epichlorohydrin-carboxymethyl cellulose, HMDI: Hexamethylene diisocyanate, CS: Chitosan. CS: Silica gel. CMMP: Fe3O4 magnetic nanoparticles (MNP) modified with carboxymethyl-β-cyclodextrin (CM-β-CD).

**Table 5:** The FT-IR typical bands of β-CD and HP-β-CD monomers.

Compound	v band (cm^−1^)	IR Vibration
β-CD	3290.61	O–H stretching vibrations
HP-β CD	3340.72	
β-CD	2924.92	C–H stretching vibrations
HP-β CD	2925.82	
β-CD	1642.90	O–H bending vibrations
HP-β CD	1645.88	
β-CD	1151.72	C–O vibration
HP-β CD	1147.82	
β-CD	1019.34	C–O–C stretching vibrations
HP-β CD	1014.01	
β-CD	857.01	α-type glycosidic bond
HP-β CD	850.69	
β-CD	-	–CH_3_ anti-symmetric vibration
HP-β CD	2968.90	
β-CD	1365.34	–CH_3_ bending vibration
HP-β CD	1369.89	
